# Anti-BACE1 and Antimicrobial Activities of Steroidal Compounds Isolated from Marine *Urechis unicinctus*

**DOI:** 10.3390/md16030094

**Published:** 2018-03-14

**Authors:** Yong-Zhe Zhu, Jing-Wen Liu, Xue Wang, In-Hong Jeong, Young-Joon Ahn, Chuan-Jie Zhang

**Affiliations:** 1 College of Chemistry and Pharmaceutical Science, Qingdao Agricultural University, Changcheng Rd, Chengyang district, Qingdao 266109, China; zhuyzh2008@163.com (Y.-Z.Z.); mail.liujingwen@gmail.com (J.-W.L.); 2 School of Pharmaceutical Sciences, Wenzhou Medical University, Wenzhou 325035, China; xinyuw001@163.com; 3 Division of Crop Protection, National Institute of Agricultural Science, Rural Development Administration, Jeollabuk-do 55365, Korea; Inhongjeong@korea.kr; 4 Department of Agricultural Biotechnology, Seoul National University, 599 Gwanak-ro, Silim-dong, Gwanak-Gu, Seoul 151742, Korea; yjahn@snu.ac.kr; 5 Department of Plant Science, University of Connecticut, 1376 Storrs Road, U-4163, Storrs, CT 06269, USA

**Keywords:** Alzheimer’s disease, antimicrobial activity, BACE1, bioassay-directed isolation, marine bioactive, *Urechis unicinctus*

## Abstract

The human β-site amyloid cleaving enzyme (BACE1) has been considered as an effective drug target for treatment of Alzheimer’s disease (AD). In this study, *Urechis unicinctus (U. unicinctus)*, which is a Far East specialty food known as innkeeper worm, ethanol extract was studied by bioassay-directed fractionation and isolation to examine its potential β-site amyloid cleaving enzyme inhibitory and antimicrobial activity. The following compounds were characterized: hecogenin, cholest-4-*en*-3-one, cholesta-4,6-*dien*-3-ol, and hurgadacin. These compounds were identified by their mass spectrometry, ^1^H, and ^13^C NMR spectral data, comparing those data with NIST/EPA/NIH Mass spectral database (NIST11) and published values. Hecogenin and cholest-4-*en*-3-one showed significant inhibitory activity against BACE1 with EC_50_ values of 116.3 and 390.6 µM, respectively. Cholesta-4,6-*dien*-3-ol and hurgadacin showed broad spectrum antimicrobial activity, particularly strongly against *Escherichia coli (E. coli)*, *Salmonella enterica (S. enterica)*, *Pasteurella multocida (P. multocida)*, and *Physalospora piricola (P. piricola)*, with minimal inhibitory concentration (MIC) ranging from 0.46 to 0.94 mg/mL. This is the first report regarding those four known compounds that were isolated from *U. unicinctus* and their anti-BACE1 and antimicrobial activity, highlighting the fact that known natural compounds may be a critical source of new medicine leads. These findings provide scientific evidence for potential application of those bioactive compounds for the development of AD drugs and antimicrobial agents.

## 1. Introduction

Alzheimer’s disease (AD) is one of the most common and devastating neurodegenerative disease associated with aging in both developed and developing countries [1,2]. There are over 46 million people suffering from AD worldwide, and this figure is expected to rise to 131.5 million by 2050 [3]. The major histopathological hallmarks of AD are the formation and accumulation of amyloid β-peptide (Aβ) in the brain [4,5]. BACE1 has been recognized as a valuable drug target for the treatment of AD due to its responsibility for initiating Aβ production [6,7]. Although there are many synthetic peptide-based inhibitors of BACE1, their comparatively large molecular weights cause difficulties, such as poor blood brain barrier and plasma membrane penetration and poor oral bioavailability, resulting in decreased pharmacological activity in vivo causing serious side effects, such as hallucinations, dizziness, and agitation [8,9]. Therefore, to date, no efficient AD medicine is available, and the development of new improved anti-AD medicine is imperative. Natural products-based BACE1 inhibitors with a low molecular weight (<700 Da) and high lipophilicity could easily cross the blood brain barrier and plasma membrane and reach the site of action (brain), following oral or transdermal administration [10,11], indicating their therapeutic potential for the treatment of AD. Plant-derived BACE1 inhibitors, such as curcuminoids [12], and terpenoids [13,14], tannins [15], and benzopyranoids [16], have been well documented [17,18] and show in vitro human BACE1 inhibitory activity [16].

Marine organisms, which represent approximately one half of the total global biodiversity, are rich sources for discovery of biofunctional compounds [19,20]. Marine bacteria and invertebrate-derived microorganisms were shown to produce promising antimicrobial compounds. For example, a cyclic peptide griseoviridin [21] and streptogramin etamycin A [22] isolated from family Streptomycetaceae showed significant antibacterial activity. A terpenoid from red sea soft corals showed a broad antibacterial effect [23]. Plenty of peptides obtained from sponge-derived microorganism with antimicrobial or antiviral activities have also been reported previously. Furthermore, numerous compounds with antihypertensive, antioxidant, anticancer, and antidiabetic biological activities, have also been discovered in the Oceans [19] and some have undergone clinical trials. To date, however, based on the published literatures, discovery of new promising marine-derived compounds for AD treatment are very limited. Thus, marine organisms as important sources for discovering potential new AD treatment drugs have drawn a lot of attention. Antibiotics have been developed and have played an important role in the treatment of bacterial infections since the last century [24]. However, extensive use of antibiotics has led to the evolution of antibiotics resistance in many bacterial species. Despite the success of the discovery of new drugs to fight against antibiotic resistance, bacterial infections remain the top leading cause of death throughout the world in 2015 [25,26]. Thus, there is an urgent need for the discovery of promising new antimicrobial compounds.

*U. unicinctus*, which is a Far East specialty food known as innkeeper worm, a member of the Echiuroidea, Urechidae, is mainly distributed in China, Korea, Japan, and Russia, and inhabits marine intertidal and subtidal zones [27]. Pharmacological studies have shown that several extracts from this species possess various biological activities, such as hypoglycemic effects [28], antithrombotic effects [29], and antitumor effects [30]. A recent study showed that activity peptide from *U. unicinctus* has positive effects on erectile function [31]. Thus, studies on *U. unicinctus* have drawn a lot of attention from the scientific community. To date, however, no study has evaluated the potential use of *U. unicinctus* for the treatment of AD. Herein, we report bioassay-directed fractionation and isolation of *U. unicinctus* extract, structural characterization of four bioactive compounds, and examine their potential BACE1 inhibitory activity and against various bacterial and fungi pathogens. To the best of our knowledge, this is the first study to report the four bioactive compounds with anti-BACE1 and antimicrobial activities isolated from marine *U. unicinctus*.

## 2. Results and Discussion

### 2.1. Identification of Isolated Bioactivie Compounds

**Figure 1:**
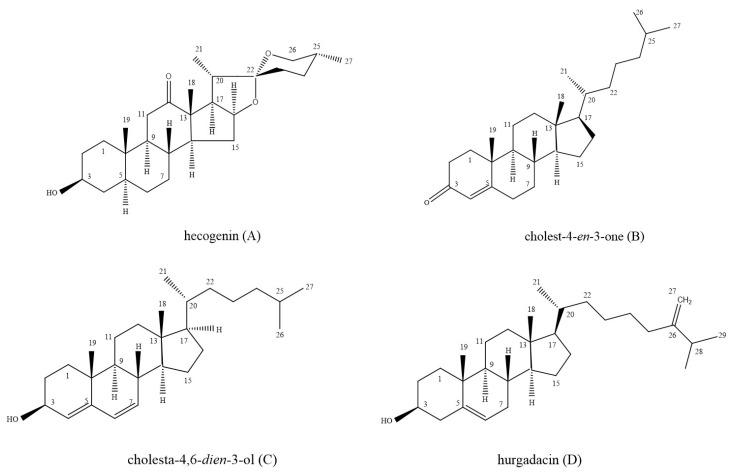
The chemical structures of isolated compounds from chloroform (**A**–**C**) and ethyl acetate (**D**) fractions of *U. unicinctus*.

On the basis of interpretation of GC-MS, ^1^H and ^13^C NMR spectral data and by comparison of corresponding data with those published values and NIST11 Mass spectral database, the two BACE1 inhibitory compounds were identified as PS-1 (hecogenin) and PS-2 (cholest-4-*en*-3-one), and the two antimicrobial compounds were PS-3 (cholesta-4,6-*dien*-3-ol) and PS-4 (hurgadacin), respectively (Figure 1). The evidence for identification were as follows: PS-1 (hecogenin), *m*/*z* (% relative intensity): 430 [M]^+^ (10), 402 (5), 358 (12), 316 (20), 273 (24), 139 (100), 126 (67), 69 (24), and with 95.8% similarity in comparison with the known hecogenin in NIST11 database (Figure S1); the typical ^1^H chemical shifts (CDCl_3_, 500 MHz): δ 3.35 (1H, m, H-3), 4.33 (1H, m, H-16), 0.80, 0.90, 1.07, and 0.78 (3H, s, H-24–27) (Figure S2); ^13^C NMR chemical shifts (CDCl_3_, 125 MHz) were shown in Figure S3 and Table 1. NMR spectrum data for hecogenin were agreement with those of Agrawal et al. 1985 [32], Jin et al. [33], and Santos and Branco [34]. PS-2 (cholest-4-*en*-3-one), *m*/*z* (% relative intensity): 384 [M]^+^ (23), 342 (12), 299 (8), 261 (20), 229 (27), 147 (24), 124 (100), 95 (21), 79 (24), and with 95.6% similarity in comparison with the known cholest-4-*en*-3-one in NIST11 database (Figure S4); the typical ^1^H chemical shifts (CDCl_3_, 500 MHz): δ 5.72 (1H, s, H-4), 0.71, 1.18 (3H, s, H-17–18), 0.92, 0.86, and 0.87 (3H, d, H-21, 26, 27) (Figure S5); ^13^C NMR (CDCl_3_, 125 MHz) were shown in Figure S6 and Table 1; NMR spectra data were identical to the data for cholest-4-*en*-3-one, as reported in previous studies [35,36,37]. PS-3 (cholesta-4,6-*dien*-3-ol), although ^1^H and ^13^C NMR analysis were not performed due to the little amount of isolated PS-3, *m*/*z* (% relative intensity) showed fragment ion at 366 (100), 247 (38), 211 (10), 91 (51), 57 (42), and 43 (67), which were consistent with the previous published MS spectrum data for cholesta-4,6-*dien*-3-ol reported by Ahire et al. [38]. In addition, the similarity of MS spectrum data between PS-3 and the known cholesta-4,6-*dien*-3-ol in NIST11 database was approximately 92.4% (Figure S7). PS-4 (hurgadacin), *m*/*z* (% relative intensity): 426 [M]^+^ (22), 314 (100), 299 (34), 271 (38), 229 (33), 145 (46), 105 (29), 55 (63), 43 (35) and with 93.3% similarity in comparison with the known hurgadacin in NIST11 database (Figure S8); the typical ^1^H chemical shifts (MeOD, 500 MHz): 3.87 (1H, dd, H-3), 4.56 (1H, d, H-27α), 4.72 (1H, d, H-27β), 0.87, 0.88, 0.95, 0.96, and 1.04 (3H, s, H-18, 19, 21, 29, 30) (Figure S9); ^13^C NMR (MeOD, 125 MHz) were shown in Figure S10 and Table 1. MS and NMR spectra data were accordance with the data for hurgadacin reported by Shaaban et al. [39].

### 2.2. In Vitro BACE-1 Inhibitory Activity of Hecogenin and Cholest-4-en-3-one

In AD treatment, BACE1 is a major drug target for inhibiting β-amyloid generation due to it being responsible for initiating β-amyloid production [6,7]. Selective phytochemicals may be applied to treat AD because they may biodegrade to nontoxic products [12]. Various compounds such as curcuminoids [12], terpenoids [13,14], and tannins [15], etc. have been tested for their inhibitory activity on the BACE1 with EC_50_s in the range of 17–359.2 µM, however, no study has been conducted regarding the BACE1 inhibitory activity of steroids. In the current study, we isolated two steroid anti-BACE1 inhibitors from marine-derived *U. unicinctus* ethanol extract and they were identified as hecogenin and cholest-4-*en*-3-one. The EC_50_ values for those two compounds were 116.3 and 390.6 µM, respectively, which were comparable to the natural product-based inhibitors aforementioned [12,13,14,15,16]. Synthetic peptide-based inhibitors (0.08–0.2 µM) [16] were more effective on inhibiting BACE1 when compared with those natural products-based inhibitors, however, due to their comparatively large molecular weights, they suffered from difficulties, such as poor blood brain barrier and plasma membrane penetration, poor oral bioavailability; thus, resulting in reduced pharmacological activity in vivo [40]. Hecogenin and cholest-4-*en*-3-one obtained in this study with the low molecular weights (430 and 384 Da, respectively) might be more efficient in crossing the blood brain barrier and plasma membrane in vivo, in comparison to synthetic compounds [10,16]. Furthermore, as they were isolated from the edible marine product, they are much safer when compared to other plant or fungi-derived compounds. It is worth noting that hecogenin and cholest-4-*en*-3-one have never been isolated from *U. unicinctus* before regarding marine-derived and non-peptidyl BACE1 inhibitors, and those might be the potential candidates to be used in prevention and/or treatment of AD.

### 2.3. Antimicrobial Activity of Cholesta-4,6-dien-3-ol and Hurgadacin

Five bacteria including *E. coli*, *S. aureus*, *S. enterica*, *M. luteus*, and *P. multocida* and three fungi including *Cytospora* sp., *P. piricola*, and *Fusarium oxysporum* f. sp. *cucumebrium* (*F. oxysporum* f. sp. *cucumebrium*) were selected for antibacterial and antifungal assays, respectively. Based on these assays (Table 2), hurgadacin displayed significant antimicrobial activity against *E. coli*, *S. enterica*, *P. multocida*, and *P. piricola* with the MIC values of 0.46, 0.94, 0.46, and 0.94 mg/mL, respectively. Antimicrobial activity was not performed for cholesta-4,6-*dien*-3-ol due to the little amount of isolation. However, subfraction C3-3-2-2, the former fraction of cholesta-4,6-*dien*-3-ol showed more potent antimicrobial activity when compared with hurgadacin, particularly strongly against *Cytospora* sp. with the MIC value of 0.46 mg/mL, indicating the potential potent antimicrobial activity of cholesta-4,6-*dien*-3-ol. In addition, both compounds showed more effective antimicrobial activity against Gram-negative bacteria than Gram-positive bacteria (Table 2 and Table S2), and less antimicrobial activity against *F. oxysporum* f. sp. *cucumebrium*. Although the MIC values of cholesta-4,6-*dien*-3-ol and hurgadacin against those strains were much lower than the common antibiotics, such as ampicillin and nystalin (the MICs ranged from 0.5 to 16 µg/mL) [41,42], they are more valuable in terms of safety and environment-friendly. Furthermore, the repeated use of antibiotic has caused the evolution of antibiotics resistance, cholesta-4,6-*dien*-3-ol, and hurgadacin would substitute and reduce the use of antibiotics.

In summary, two BACE1 inhibitory compounds, hecogenin and cholest-4-*en*-3-one, and two antimicrobial compounds, cholesta-4,6-*dien*-3-ol and hurgadacin, were first isolated from marine-derived *U. unicinctus* and showed BACE1 inhibitory activity (EC_50_, 116.3 and 390.6 µM, respectively) and broad spectrum antimicrobial activity, particularly strongly against *E. coli*, *S. enterica*, and *P. multocida*, respectively. Although they are known compounds, no studies have elucidated their anti-BACE1 and antimicrobial activity to date, highlighting the fact that known natural compound may be a critical source of new medicine leads.

## 3. Materials and Methods

### 3.1. Experimental Materials and Chemicals

*U. unicinctus* was purchased from a local aquatic market (Noryangjin, Korea) August 2015 in Seoul, Republic of Korea. It was mainly harvested from a coastal intertidal mudflat in Incheon, Republic of Korea. Recombinant human BACE1 and fluorogenic peptide substrate (FPS) Mca-SEVNLDAEFRK (Dnp) RR-NH2 were purchased from R & D System (Minneapolis, MN, USA). The tested microorganisms including bacteria, *E. coli* (Gram-negative), *S. aureus* (Gram-positive), *S. enterica* (Gram-negative), *M. luteus* (Gram-positive), and *P. multocida* (Gram-negative), and fungi, *Cytospora* sp., *P. piricola*, and *F. oxysporum* f. sp. *cucumebrium* were obtained from School of Life Science and School of Agronomy and Plant Protection, Qingdao Agricultural University (Qingdao, China). Thin layer chromatography (TLC) GF254 was purchased from Shenhai Silicon Material Co. (Qingdao, China). Resazurin was from Sigma-Aldrich (Shanghai, China). Sephadex LH 20 was purchased from Merck (Darmstadt, Germany). Potato dextrose agar (PDA), potato dextrose broth (PDB), and lysogeny broth (LB) were purchased from Qingdao Hope Bio-Technology (Qingdao, China). All of the other chemicals and reagents that were used in this study were of analytical-grade quality and are available commercially.

### 3.2. Bioassay-Directed Isolation of U. unicincuts Extracts

**Figure 2:**
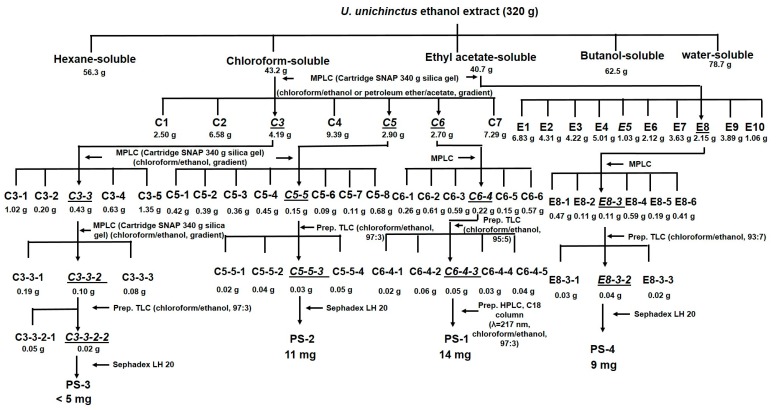
Bioassay-directed isolation scheme of BACE1 inhibitory and antimicrobial activity compounds from *U. unicinctus*.

An approach of bioassay-directed isolation was used to determine the most bioactive constituents from *U. unicinctus* extracts. *U. unicinctus* (320 g) was homogenized in a mortar with anhydrous ethanol (1:5, *w*/*v*), and the mixtures were extracted (three times) at 20 °C for 24 h and filtered. The combined filtrates were concentrated by rotary evaporation at 40 °C. The crude extract was sequentially partitioned into hexane- (56.3 g), chloroform- (43.2 g), ethyl acetate- (40.7 g), butanol- (62.5 g), and water-soluble (78.7 g) portions (Figure 2). To isolate the bioactive constituents, 0.5–2.0 mg/mL of each fraction was tested in a Fluorescence Resonance Energy Transfer (FRET)-based enzyme assay and antimicrobial active assay as described below. Chloroform- (43.2 g) and ethyl acetate-soluble fractions (40.7 g) were found to be the most active fractions in those two assays. In FRET-based enzyme assay, the chloroform-soluble fraction showed 65.1–79.8% of inhibitory activity against BACE1, followed by ethyl acetate- (40.0–50.3%), butanol- (30.7–40.4%), hexane- (24.3–39.1%), and water-soluble fraction (13.2–17.1%), in comparison with positive reference curcumin (88.0–98.5%) (Table 3). In antimicrobial activity assay, chloroform- and ethyl acetate-soluble fractions were the most potent constituents to suppress the growth of those tested microorganisms, except for *M. luteus* (Table 4). Thus, those two active fractions were further subjected to a medium pressure liquid chromatography (MPLC) using an Isolera apparatus equipped with an ultraviolet (UV) detector at 254 and 365 nm and a SNAP column cartridge (340 g silica gel) eluted with the appropriate gradient of chloroform/ethanol or petroleum ether/acetate to afford the most active subfractions C3 (4.189 g), C5 (0.151 g), C6 (2.703 g), and E8 (2.147 g) (Figure 2). The subfractions were monitored by TLC on silica gel plates and visualized by spraying the plates with 2% sulfuric acid. The subfractions with similar *R*_f_ values on the TLC plants were pooled. Those active subfractions C3, C5, C6, and E8 were continuously separated by the same silica gel column chromatography, as described previously, eluted with the appropriate gradient of chloroform/ethanol, and further purified by preparative TLC or Agilent 1100 HPLC (Agilent Technologies, Santa Clara, CA, USA) or by column chromatography over Sephadex LH 20. The results of BACE1 inhibitory activity and antimicrobial activity for those bioactive subfractions were summarized in Tables S1 and S2. During the isolation, only the most bioactive subfractions were further isolated. Ultimately, the two most bioactive compounds, PS-1 (14 mg) and PS-2 (11 mg), in FRET-based enzyme assay and the two most bioactive compounds, PS-3 (<5 mg) and PS-4 (9 mg), in antimicrobial active assay were obtained (Figure 2).

### 3.3. Identification of Isolated Bioactive Compounds

The four bioactive compounds were identified by GC-MS and NMR spectral data. GC-MS analysis was conducted using an Agilent 5975B inert series GC/MS (Agilent Technologies, Santa Clara, CA, USA), equipped with a J&W DB-5MS column (30 m × 320 μm × 0.25 μm) and operated in electron ionization (EI) mode at 70 eV with a scan range of 40–500 *m*/*z*. Helium was used as the carrier gas (1.5 mL/min). The temperature of the inlet was set to 230 °C. The column temperature initiated at 50 °C for 2 min, and then was programmed to rise to 120 °C at the rate of 2 °C/min, then to 160 °C at the rate of 10 °C/min, and finally to 220 °C at the rate of 20 °C/min, and then maintained for 5 min at the level of 220 °C. ^1^H and ^13^C NMR spectra were recorded in CDCl_3_ or MeOD on AVANCE III 500 MHz spectrometer (Bruker, Switzerland) at 500 and 125 MHz using tetramethylsilane as an internal standard. The chemical shifts are given in δ (ppm). Their spectral data were then compared with published values and further confirmed using NIST/EPA/NIH Mass spectral database (NIST11) with NIST MS search program v.2.0 g. Regarding PS-3, due to the little amount of isolation, only GC-MS analysis was conducted.

### 3.4. Fluorescence Resonance Energy Transfer (FRET)-Based Enzyme Assay

To assess BACE1 inhibitory activity of isolate constituents from *U. unicinctus* extract, FRET-based enzyme assay was used [12,43,44]. Reaction mixtures containing 47.3 µL of 50 mM sodium acetate (PH 4.5), 0.75 µL of a 2.5 µg/µL fluorogenic peptide substrate, 1 µL of 0.5 µg/µL recombinant human BACE-1, and the isolated subfractions (12.5–100 mg/mL) in 2% DMSO were incubated in darkness for 60 min at 25 °C followed by adding 16.6 µL of 2.5 M sodium acetate to terminate the reaction prior to measurements of fluorescence intensity. The measurements were performed using a SpectraMAX Gemini XS plate reader (Sunnyvale, CA, USA) at 320 nm excitation and 405 nm emission. Curcumin, which is an effective BACE1 inhibitor, was used as positive reference [12]. Negative control (without tested subfractions) and blank control (without BACE1) were also included. The inhibition percentage was calculated with the following Equation (1):

(1) $$\%\;\mathrm{inhibition}=(1-\frac{F_{s60}-F_{s0}}{F_{c60}-F_{c0}})\times 100$$

where *F_S_*_60_ and *F_S_*_0_ represent the fluorescence intensity of tested subfractions at 60 min and 0, and *F_C_*_60_ and *F_C_*_0_ represent the fluorescence intensity of control at 60 min and 0, respectively [43]. All of the experiments were conducted in triplicate.

### 3.5. Antimicrobial Activity Assay

Antimicrobial activity assay, including antifungal and antibacterial activity assays, was performed in 96-well sterilized microplates using a 2-fold dilution method to determine the MIC [45,46]. Aforementioned fungi and bacteria were grown in potato dextrose broth (PDB) and lysogeny broth (LB) medium, respectively, for 24–72 h prior to the experiments. Each fungus and bacterium were incubated at their optimum growth temperature to achieve 1 × 10^4^ spores/mL and 1 × 10^5^ colony-forming units/mL, respectively. In a 96-well plate, an aliquot of PDB (85 µL) or LB (80 µL) broth containing strains was distributed. After that, the test compounds were 2-fold diluted in DMSO, and their final concentrations ranged from 15 to 0.23 mg/mL and 3.75 to 0.23 mg/mL based on the amount of obtained isolates. The wells containing tested strains and diluted compounds (15 µL) were incubated at 27 °C (72 h) for fungi and 36 °C (24 h) for bacteria. The wells containing strains culture suspension and DMSO were run as negative control and were incubated under same conditions as those used for bacteria and fungi cultures. With regard to antibacterial activity assay, after incubation, 5 µL (1.0 mg/mL) of resazurin dye was added to each well, and further incubated for 2–4 h for the observation of color change. The wells with no color change (remained blue) were scored above the MIC value. As the little amount of PS-3 isolated, antimicrobial activity assay was not performed. In the case of antifungal activity assay, the level of growth inhibition was determined by comparing absorbance measurements (530 nm) taken at 72 h with the reading at 0 h and subtracting control backgrounds (DMSO). All of the experiments were conducted in triplicate.

### 3.6. Data Analysis

All of the results are presented as mean ± standard error (SE) of three replicates. The effective concentration of tested compound causing a 50% inhibition of BACE1 activity (EC_50_) was estimated by fitting the data to the four-parameter log-logistic dose-response model using GraphPad Prism 5.1 software (GraphPad software, San Diego, CA, USA). There was no evidence of lack-of-fit of the model.

## 4. Conclusions

Hecogenin and cholest-4-*en*-3-one as non-peptidyl BACE1 inhibitors, are potential therapeutics and promising compounds for the prevention and/or treatment of AD. Cholesta-4,6-*dien*-3-ol and hurgadacin may be beneficial for the development of an antimicrobial agent or a food preservative. In addition, cholesta-4,6-*dien*-3-ol may be exploited as an agricultural fungicide due to its strong antifungal activity toward *Cytospora* sp. and *P. piricola*. The future study would be focused on the cytotoxicity and cell membrane penetration (hecogenin and cholest-4-*en*-3-one) and the related animal experiment, as well as the mechanisms of action of antimicrobials (cholesta-4,6-*dien*-3-ol and hurgadacin) and their applications. 

## Figures and Tables

**Table 1:** ^13^C NMR spectral data for PS-1, PS-2, and PS-4 isolated from *U. unicinctus* in CDCl_3_ (δ in ppm).

Carbon Atom No.	PS-1 (Hecogenin)	Reference [34]	PS-2 (Cholest-4-*en*-3-one)	Reference [37]	PS-4 (Hurgadacin)	Reference [39]
1	36.5	36.6	35.7	35.7	36.3	37.2
2	31.2	31.5	33.9	34.0	30.9	31.6
3	70.8	71.0	199.5	199.6	71.0	71.7
4	37.8	38.0	123.7	123.7	41.6	42.3
5	44.6	44.7	171.0	171.6	140.8	140.7
6	28.7	28.5	32.9	33.0	121.0	121.6
7	28.3	31.6	32.0	32.1	31.8	31.9
8	34.3	34.4	35.6	35.7	31.6	31.9
9	55.1	55.6	53.8	53.8	50.3	50.1
10	36.1	36.2	38.6	38.6	37.1	36.5
11	37.8	38.0	21.0	21.0	21.1	21.2
12	213.6	213.7	39.6	39.6	39.8	39.8
13	55.5	55.2	42.3	42.4	42.1	42.3
14	55.8	55.9	55.8	55.9	56.7	56.7
15	31.5	31.6	24.1	24.2	23.9	24.3
16	79.2	79.3	28.1	28.2	27.9	28.2
17	53.5	53.6	56.1	56.1	56.0	56.0
18	16.0	16.1	11.9	12.0	10.9	11.2
19	11.9	12.0	17.3	17.4	18.5	19.4
20	42.1	42.3	76.7	33.8	35.6	35.7
21	13.2	13.3	18.6	18.6	17.8	18.7
22	109.2	109.3	36.1	36.1	34.6	34.7
23	31.4	31.3	23.8	23.8	28.8	28.2
24	30.1	28.9	39.5	39.5	29.4	31.6
25	31.1	30.3	28.0	28.1	30.7	31.0
26	66.8	67.0	22.5	22.6	156.4	156.8
27	17.1	17.2	22.8	22.8	105.5	105.9
28	-	-	-	-	33.5	33.8
29	-	-	-	-	20.8	22.0
30	-	-	-	-	20.9	21.8

NMR analysis was not performed for PS-3 (cholesta-4,6-*dien*-3-ol) due to the little amount of isolation.

**Table 2:** Minimal inhibitory concentrations (MIC) (mg/mL) of C3-3-2-2 and hurgadacin against bacterial and fungal.

Materials	*E. coli* (G−)	*S. aureus* (G+)	*S. enterica* (G−)	*M. luteus* (G+)	*P. multocida* (G−)	*Cytospora* sp.	*P. piricola*	*F. oxysporum* f. sp. *cucumebrium*
C3-3-2-2 ^a^	0.46	- ^b^	0.46	-	0.46	0.46	0.94	-
hurgadacin	0.46	3.75	0.94	>3.75	0.46	>3.75	0.94	>3.75

The tested concentrations ranged from 3.75 to 0.23 mg/mL for C3-3-2-2 and hurgadacin. ^a^ the former fraction of cholesta-4,6-*dien*-3-ol; ^b^ The strains were not tested as the unsatisfied antimicrobial activity displayed in the former fractions.

**Table 3:** BACE1 inhibitory activity (mg/mL, ±standard error, three replicates) of fractions that were obtained from *U. unicinctus* crude ethanol extract.

Materials (Soluble Fraction)	% Inhibition		
	0.5 (mg/mL)	1.0 (mg/mL)	2.0 (mg/mL)
Curcumin	88.03 ± 0.34	93.21 ± 0.52	98.51 ± 0.12
Hexane	24.3 ± 0.69	36.9 ± 0.13	39.1 ± 0.22
Chloroform	65.1 ± 0.21	75.2 ± 0.16	79.8 ± 0.06
Ethyl acetate	40.0 ± 0.10	48.3 ± 0.11	50.3 ± 0.12
Butanol	30.7 ± 0.07	37.4 ± 0.18	40.4 ± 0.25
Water	13.2 ± 0.28	14.9 ± 0.26	17.1 ± 0.11

**Table 4:** Antimicrobial activity (3 replicates) of fractions obtained from *U. unicinctus* crude ethanol extract.

Materials (Soluble Fraction)	Concentration (mg/mL)	*E. coli* (G−)	*S. aureus* (G+)	*S. enterica* (G−)	*M. luteus* (G+)	*P. multocida* (G−)	*Cytospora* sp.	*P. piricola*	*F. oxysporum* f. sp. *cucumebrium*
Hexane	7.5	-	-	-	-	-	-	-	-
	15	+	-	+	-	+	-	-	-
Chloroform	7.5	+	+	+	-	+	+	+	+
	15	+	+	+	-	+	+	+	+
Ethyl acetate	7.5	+	-	+	-	+	+	+	+
	15	+	+	+	-	+	+	+	+
Butanol	7.5	-	-	-	-	-	-	-	-
	15	-	-	-	-	-	-	-	-
Water	7.5	-	-	-	-	-	-	-	-
	15	-	-	-	-	-	-	-	-

G−, Gram-negative; G+, Gram-positive; +, strong inhibitory activity; -, no antibacterial activity.

## Supplementary Materials

The following are available online at http://www.mdpi.com/1660-3397/16/3/94/s1, Figure S1: Known hecogenin in NIST11 database (A) and EI-MS (70 ev) spectrum of PS-1 (B), Figure S2: ^1^H NMR (CDCl_3_, 500 MHz) spectrum of PS-1, Figure S3: ^13^C NMR (CDCl_3_, 125 MHz) spectrum of PS-1, Figure S4: Known cholest-4-*en*-3-one in NIST11 database (A) and EI-MS (70 ev) spectrum of PS-2 (B), Figure S5: ^1^H NMR (CDCl_3_, 500 MHz) spectrum of PS-2, Figure S6: ^13^C NMR (CDCl_3_, 125 MHz) spectrum of PS-2, Figure S7: Known cholesta-4,6-*dien*-3-ol in NIST11 database (A) and EI-MS (70 ev) spectrum of PS-3 (B), Figure S8: Known hurgadacin in NIST11 database (A) and EI-MS (70 ev) spectrum of PS-4 (B), Figure S9: ^1^H NMR (MeOD, 500 MHz) spectrum of PS-4, Figure S10: ^13^C NMR (MeOD, 125 MHz) spectrum of PS-4, Table S1: Summary of BACE1 inhibitory activity (% inhibition, ±standard error) of chloroform-soluble fractions, Table S2: Summary of antimicrobial inhibitory activity of chloroform- and ethyl acetate-soluble fractions.

## Abbreviations

AD	Alzheimer’s disease
BACE1	β-site amyloid cleaving enzyme
FRET	fluorescence resonance energy transfer
HPLC	high-performance liquid chromatography
LB	lysogeny broth
MIC	minimal inhibitory concentration
MPLC	medium pressure liquid chromatography
MS	mass spectrometry
NMR	nuclear magnetic resonance spectroscopy
PDA	potato dextrose agar
PDB	potato dextrose broth

## References

[1] Kalaria R., Maestre G.E., Arizaga R., Friedland R.P., Galasko D., Hall K., Luchsinger J.A., Ogunniyi A., Perry E.K., Potocnik F. (2008). Alzheimer’s disease and vascular dementia in developing countries: Prevalence, management, and risk factors. Lancet Neurol..

[2] Anand P., Singh B., Singh N. (2012). A review on coumarins as acetylcholinesterase inhibitors for Alzheimer’s disease. Bioorg. Med. Chem..

[3] Prince M., Wimo A., Guerchet M., Ali G., Wu Y., Prina M. (2015). World Alzheimer Report 2015: The Global Impact of Dementia: An Analysis of Prevalence, Incidence, Cost and Trends.

[4] Vassar R., Bennett B.D., Babu-Kahn S. (1999). β-secretase cleavage of Alzheimer’s amyloid precursor protein by the transmembrane aspartic protease BACE. Science.

[5] Hardy J., Selkoe D.J. (2002). The amyloid hypothesis of Alzheimer’s disease: Progress and problems on the road to therapeutics. Science.

[6] Vassar R., Kovacs D.M., Yan R., Wong P.C. (2009). The β-secretase enzyme BACE in health and Alzheimer’s disease: Regulation, cell biology, function, and therapeutic potential. J. Neurosci..

[7] De Strooper B., Vassar R., Golde T. (2010). The secretases: Enzymes with therapeutic potential in Alzheimer disease. Nat. Rev. Neurol..

[8] Molinuevo J.L., Lladó A., Rami L. (2005). Memantine: Targeting glutamate excitotoxicity in Alzheimer’s disease and other dementias. Am. J. Alzheimers Dis. Other Dement..

[9] Thompson S., Lanctôt K.L., Herrmann N. (2004). The benefits and risks associated with cholinesterase inhibitor therapy in Alzheimer’s disease. Expert Opin. Saf..

[10] Broadwell R.D., Sofroniew M.V. (1999). Serum Proteins Bypass the Blood-Brain Fluid Barriers for Extracellular Entry to the Central Nervous System. Exp. Neurol..

[11] Jeon S.Y., Bae K., Seong Y.H., Song K.S. (2003). Green tea catechins as a BACE1 (β-secretase) inhibitor. Bioorg. Med. Chem. Lett..

[12] Wang X., Kim J.R., Lee S.B., Kim Y.J., Jung M.Y., Kwon H.W., Ahn Y.J. (2014). Effects of curcuminoids identified in rhizomes of *Curcuma longa* on BACE-1 inhibitory and behavioral activity and lifespan of Alzheimer’s disease Drosophila models. BMC Complement. Altern. Med..

[13] Mukherjee P.K., Kumar V., Mal M., Houghton P.J. (2007). Acetylcholinesterase inhibitors from plants. Phytomedicine.

[14] Choi Y.H., Yon G.H., Hong K.S., Yoo D.S., Choi C.W., Park W.K., Kong J.Y., Kim Y.S., Ryu S.Y. (2008). In vitro BACE-1 inhibitory phenolic components from the seeds of *Psoralea corylifolia*. Planta Med..

[15] Youn K., Jun M. (2013). In vitro BACE1 inhibitory activity of geraniin and corilagin from *Geranium thunbergii*. Planta Med..

[16] Marumoto S., Miyazawa M. (2010). β-secretase inhibitory effects of furanocoumarins from the root of *Angelica dahurica*. Phytother. Res..

[17] Orhan I.E. (2012). Current concepts on selected plant secondary metabolites with promising inhibitory effects against enzymes linked to Alzheimer’s disease. Curr. Med. Chem..

[18] Jung H.A., Lee E.J., Kim J.S., Kang S.S., Lee J.H., Min B.S., Choi J.S. (2009). Cholinesterase and BACE1 inhibitory diterpenoids from *Aralia cordata*. Arch. Pharm. Res..

[19] Harnedy P.A., FitzGerald R.J. (2012). Bioactive peptides from marine processing waste and shellfish: A review. J. Funct. Foods.

[20] Schinke C., Martins T., Queiroz S.C.N., Melo I.S., Reyes F.G.R. (2017). Antibacterial compounds from marine bacteria. J. Nat. Prod..

[21] Lin Q., Liu Y. (2010). A new marine microorganism strain L0804: Taxonomy and characterization of active compounds from its metabolite. World J. Microbiol. Biotechnol..

[22] Haste N.M., Perera V.R., Maloney K.N., Tran D.N., Jensen P., Fenical W., Nizet V., Hensler M.E. (2010). Activity of the streptogramin antibiotic etamycin against methicillin-resistant *Staphylococcus aureus*. J. Antibiot..

[23] Gomaa M.N., Soliman K., Ayesh A., El-Wahed A.A., Hamza Z., Mansour H.M., Khalifa S.A.M., Ali H.B.M., El-Seedi H.R. (2016). Antibacterial effect of the red sea soft coral *Sarcophyton trocheliophorum*. Nat. Prod. Res..

[24] Liu J., Jung J.H., Liu Y. (2016). Antimicrobial compounds from marine invertebrates-derived microorganisms. Curr. Med. Chem..

[25] Procopio R.E., De L., Da Silva I.R., Martins M.K., De Azevedo J.C., De Araujo J.M. (2012). Antibiotics produced by *Streptomyces*. Braz. J. Infect. Dis..

[26] Passari A.K., Chandra P., Leo V.V., Mishra B.K., Singh B.P. (2017). Production of potent antimicrobial compounds from *Streptomyces cyaneofuscatus* associated with fresh water sediment. Front. Microbiol..

[27] Li F.L., Wang W., Zhou H. (1994). Studies on the echiurans (echiura) of the yellow sea (Huanghai) and Bohai sea. J. Ocean Univ. Qingdao.

[28] Yuan C., Liu P., Han X., Cui Q. (2015). Hypoglycemic effects of glycosaminoglycan from *Urechis unicinctus* in diabetic mice. J. Med. Food.

[29] Bi Q., Han B., Feng Y., Jiang Z., Yang Y., Liu W. (2013). Antithrombotic effects of a newly purified fibrinolytic protease from *Urechis unicinctus*. Thromb. Res..

[30] Sung W.S., Park S.H., Lee D.G. (2008). Antimicrobial effect and membrane-active mechanism of urechistachykinins, neuropeptides derived from *Urechis unicinctus*. FEBS Lett..

[31] Kim K.S., Bae W.J., Kim S.J., Kang K.-H., Kim S.-K., Cho H.J., Hong S.-H., Lee J.Y., Kim S.W. (2016). Improvement of erectile dysfunction by the active peptide from *Urechis unicinctus* by high temperature/pressure and ultra—Wave assisted lysis in Streptozotocin Induced Diabetic Rats. Int. Braz. J. Urol..

[32] Agrawal P.K., Jain D.C., Gupta R.K., Thakur R.S. (1985). Carbon-13 NMR spectroscopy of steroidal sapogenins and steroidal saponins. Phytochemistry.

[33] Jin J.M., Liu X.K., Yang C.R. (2003). Three new hecogenin glycosides from fermented leaves of *Agave Americana*. J. Asian Nat. Prod. Res..

[34] Santos J.D.G., Branco A. (2014). GC-MS characterisation of *Sapogenins* from sisal Waste and a method to isolate pure hecogenin. Bioresources.

[35] Chiang Y.-B., Ismail W., Muller M., Fuchs G. (2007). Initial steps in the anoxic metabolism of cholesterol by the denitrifying *Sterolibacterium denitrificans*. J. Biol. Chem..

[36] Liu Y.C., Chen G.Y., Ge F.L., Li W., Zeng L.H., Cao W.G. (2011). Efficient biotransformation of cholesterol to androsta-1,4-diene-3,17-dione by a newly isolated actinomycete *Gordonia neofelifaecis*. World Microbiol. Biotechnol..

[37] Wu K., Li W., Song J., Li T. (2015). Production, purification, and identification of cholest-4-en-3-one produced by cholesterol oxidase from *Rhodococcus* sp. in aqueous/organic biphasic system. Biochem. Insights.

[38] Ahire J.J., Mokashe N.U., Chaudhari B.L. (2014). Cholesterol biotransformation to cheolesta-4,6-dien-3-ol and effect of assimilation on adhesion properties of *Lactobacillus helveticus* CD6. J. Microbiol. Biotechnol. Food Sci..

[39] Shaaban M., Shaaban K.A., Ghani M.A. (2013). Hurgadacin: A new steroid from Sinularia polydactyla. Steroids.

[40] Vassar R. (2014). BACE1 inhibitor drugs in clinical trials for Alzheimer’s disease. Alzheimers Res. Ther..

[41] Salmon S.A., Watts J.L. (2000). Minimum inhibitory concentration determinations for various antimicrobial agents against 1570 bacterial isolates from Turkey Poults. Avian Dis..

[42] Tian S.-Z., Pu X., Luo G., Zhao L.-X., Xu L.-H., Li W.-J., Luo Y. (2013). Isolation and characterization of new *p*-terphenyls with antifungal, antibacterial, and antioxidant activities from halophilic actinomycete *Nocardiopsis gilva* YIM 90087. J. Agric. Food Chem..

[43] Lv L., Yang Q.Y., Zhao Y., Yao C.S., Sun Y., Yang E.J., Song K.S., Mook-Jung I., Fang W.S. (2008). BACE1 (β-secretase) inhibitory chromone glycosides from *Aloe vera* and *Aloe nobilis*. Planta Med..

[44] Wang X., Perumalsamy H., Kwon H.W., Na Y.-E., Ahn Y.J. (2015). Effects and possible mechanisms of action of acacetin on the behavior and eye morphology of *Drosophila* models of Alzheimer’s disease. Sci. Rep..

[45] Elshikh M., Ahmed S., Funston S., Dunlop P., McGaw M., Marchant R., Banat I.M. (2016). Resazurin-based 96-well plate microdilution method for the determination of minimum inhibitory concentration of biosurfactants. Biotechnol. Lett..

[46] Jorgensen J.H., Ferraro M.J. (2009). Antimicrobial susceptibility testing: A review of general principles and contemporary practices. Clin. Infect. Dis..

