# Supplementary material for: Anti-BACE1 and Antimicrobial Activities of Steroidal Compounds Isolated from Marine Urechis unicinctus
Source: Mar Drugs. 2018 Mar 14;16(3):94. doi: 10.3390/md16030094 (PMC5867638; doi:10.3390/md16030094)
Supplement: Supplementary file 1 [file marinedrugs-16-00094-s001.docx]

**Supplentary materials**

**Anti-BACE1 and Antimicrobial Activities of Steroidal Compounds Isolated from Marine *Urechis unichinctus***

Yong-Zhe Zhu^†^, Jing-Wen Liu^†^, Xue Wang^‡^, In-Hong Jeong^§^, Young-Joon Ahn^⊥^, Chuan-Jie Zhang^║,*^

^†^College of Chemistry and Pharmaceutical Science, Qingdao Agricultural University, Changcheng Rd, Chengyang district, Qingdao, Shandong, 266109, China

^‡^School of Pharmaceutical Sciences, Wenzhou Medical University, Wenzhou, Zhejiang, 325035, China

^§^Division of Crop Protection, National Institute of Agricultural Science, Rural Development Administration, Jeollabuk-do 55365, Republic of Korea

^⊥^Department of Agricultural Biotechnology, Seoul National University, 599 Gwanak-ro, Silim-dong, Gwanak-Gu, Seoul, 151742, Republic of Korea

^║^Department of Plant Science, University of Connecticut, 1376 Storrs Road, U-4163, Storrs, CT 06269, United State


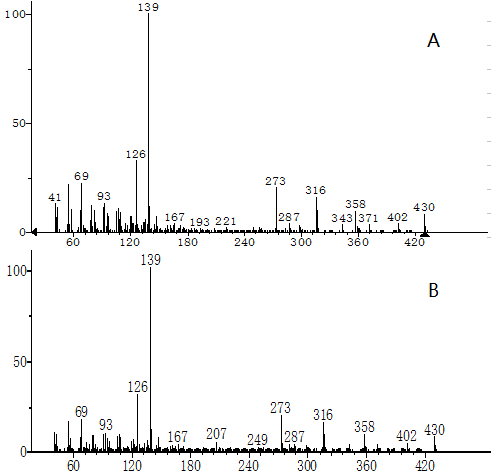


**Figure S1.** Known hecogenin in NIST11 database (A) and EI-MS (70 ev) spectrum of PS-1 (B).


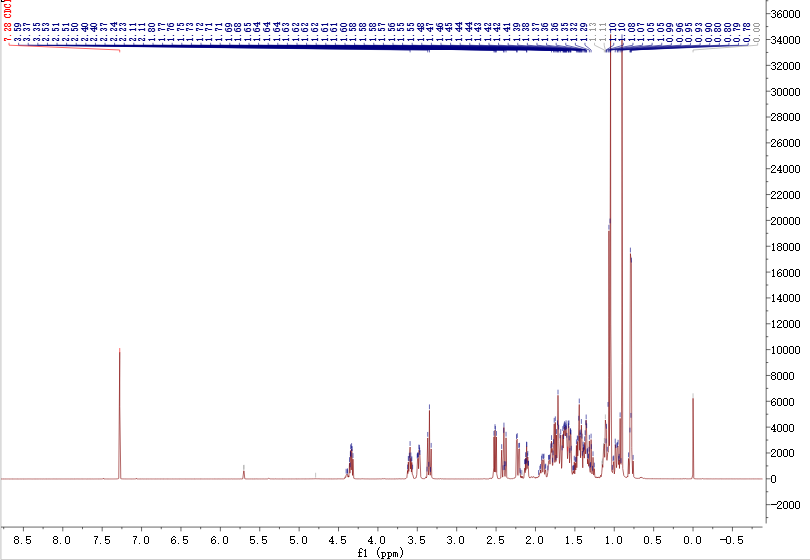


**Figure S2.** ^1^H NMR (CDCl_3_, 500 MHz) spectrum of PS-1.


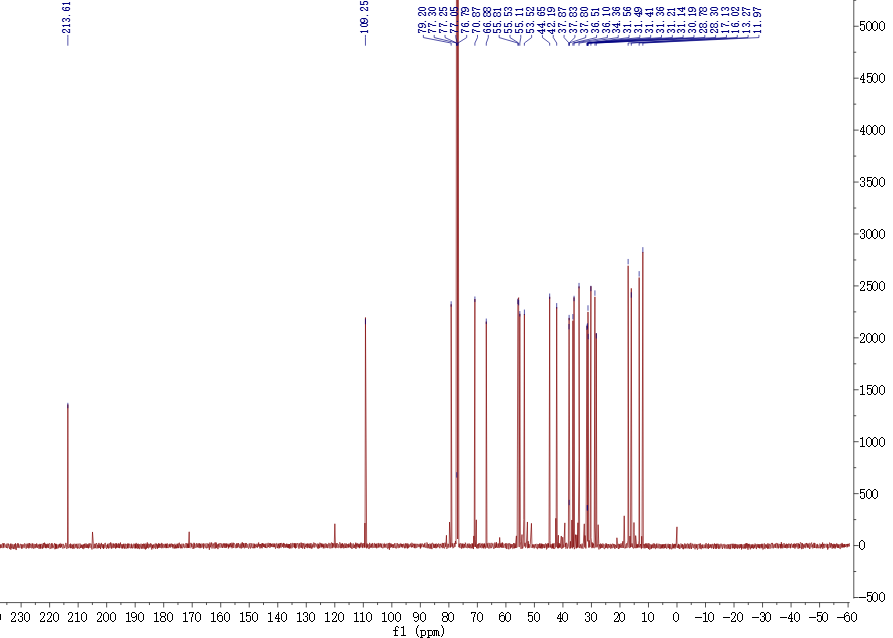


**Figure S3.** ^13^C NMR (CDCl_3_, 125 MHz) spectrum of PS-1.


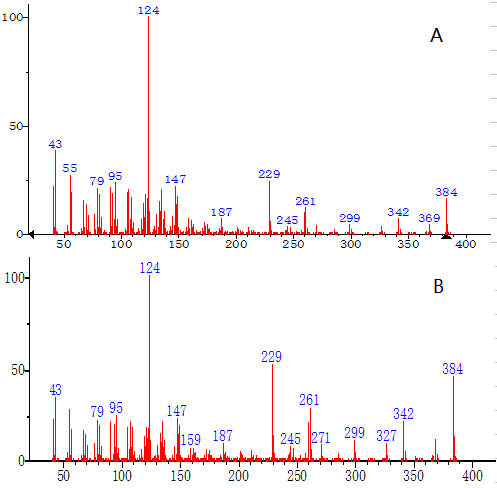
 **S3 Figure S4.** Known cholest-4-*en*-3-one in NIST11 database (A) and EI-MS (70 ev) spectrum of PS-2 (B).


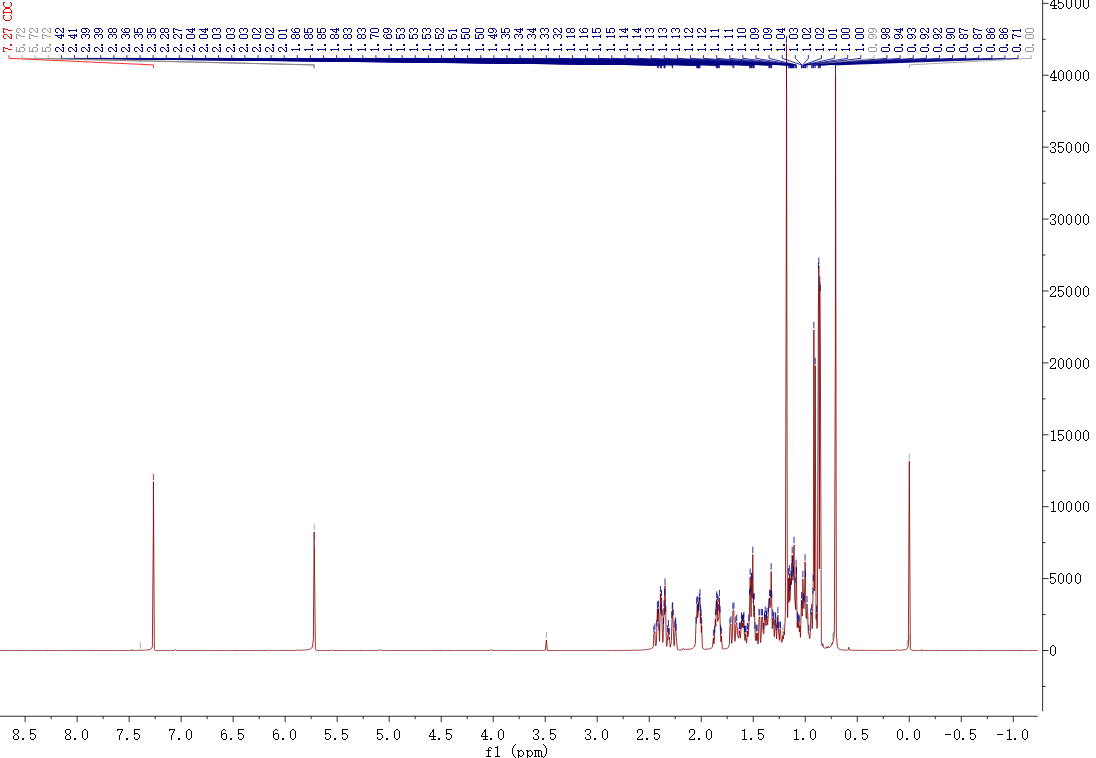
 **Figure S5.** ^1^H NMR (CDCl_3_, 500 MHz) spectrum of PS-2.


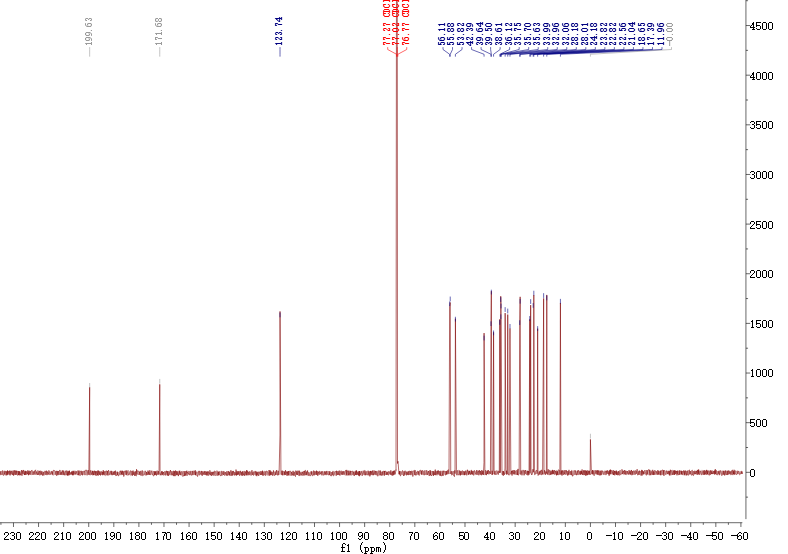


**Figure S6.** ^13^C NMR (CDCl_3_, 125 MHz) spectrum of PS-2.


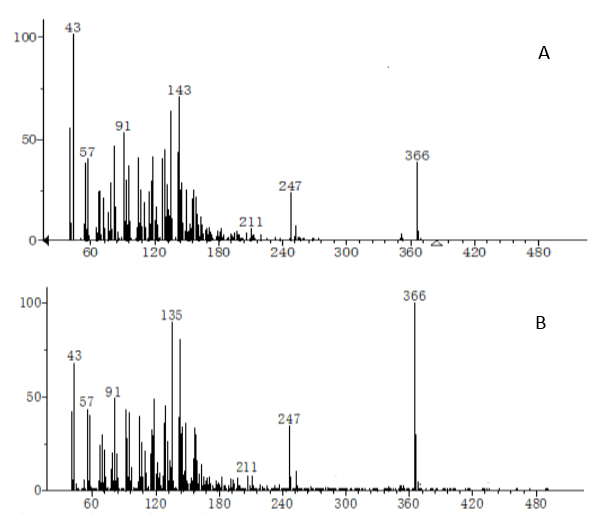


**Figure S7.** Known cholesta-4,6-*dien*-3-ol in NIST11 database (A) and EI-MS (70 ev) spectrum of PS-3 (B).


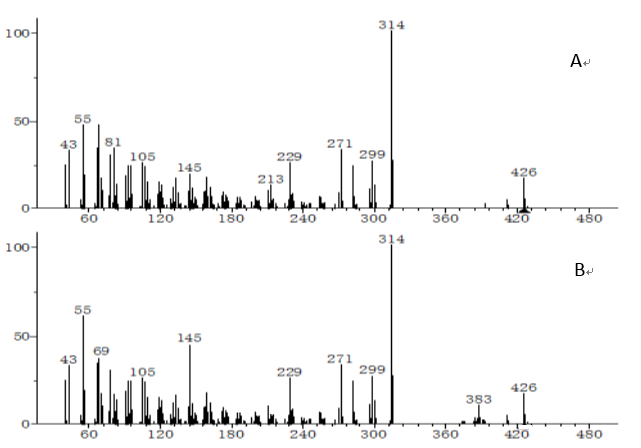


**Figure S8.** Known hurgadacin in NIST11 database (A) and EI-MS (70 ev) spectrum of PS-4 (B).


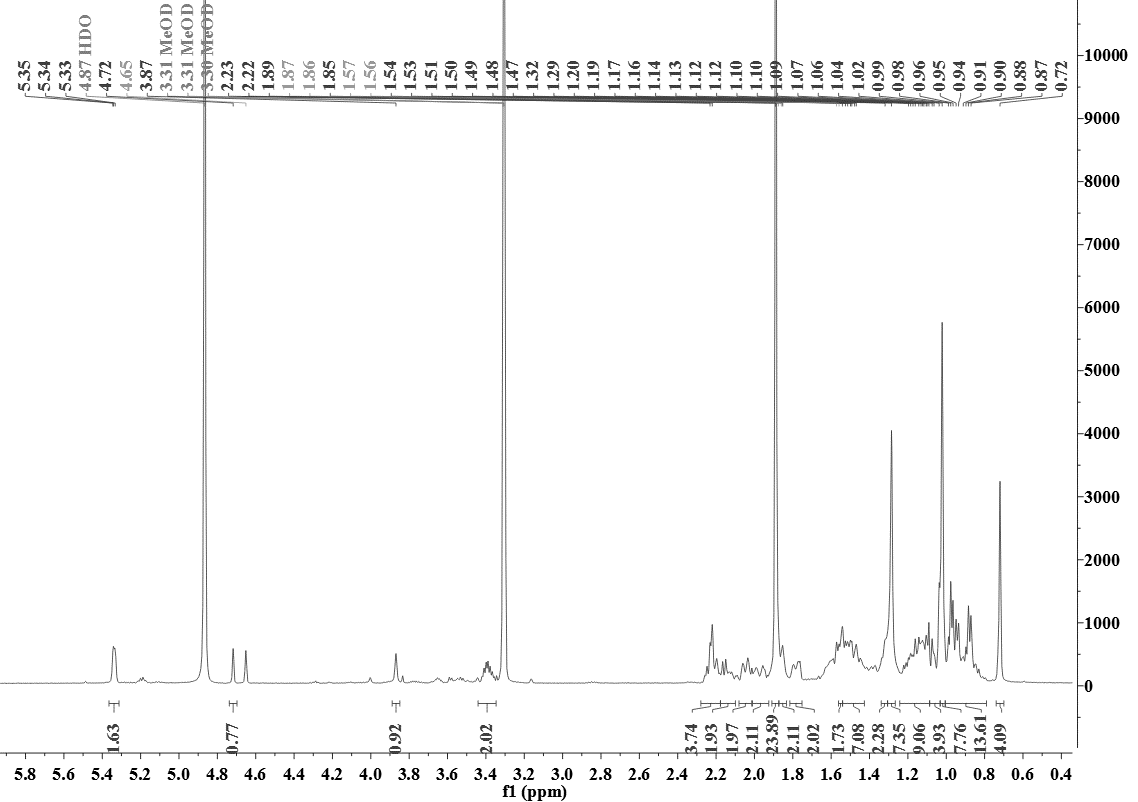
 **Figure S9.** ^1^H NMR (MeOD, 500 MHz) spectrum of PS-4.
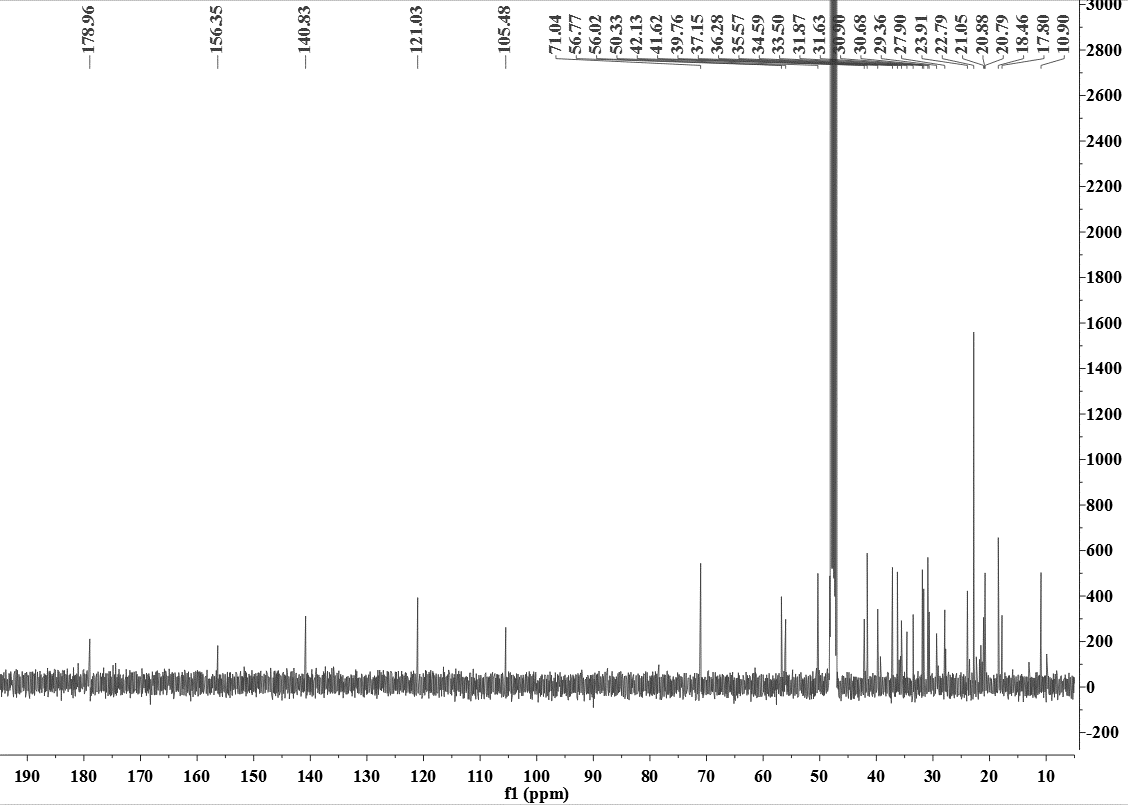


**Figure S10.** ^13^C NMR (MeOD, 125 MHz) spectrum of PS-4.

**Table S1.** Summary of BACE1 inhibitory activity (% inhibition, ± standard error) of chloroform-soluble fractions.

| Materials | % Inhibition | | | |
| --- | --- | --- | --- | --- |
|  | 0.25 (mg/mL) | 0.5 (mg/mL) | 1.0 (mg/mL) | 2.0 (mg/mL) |
| Curcuminoids ^a^ | -^b^ | 88.03±0.34 | 93.21±0.52 | 98.51±0.12 |
| C1 | - | 0 | 0 | 0 |
| C2 | - | 4.32±0.15 | 6.94±2.27 | 10.07±1.21 |
| C3 | - | 40.12±0.18 | 46.65±0.26 | 52.11±0.47 |
| C4 | - | 41.21±1.79 | 46.58±1.64 | 61.05±0.22 |
| C5* | - | 75.06±0.72 | 87.47±1.12 | 94.15±1.43 |
| C6* | - | 90.03±0.51 | 94.12±0.98 | 100.00±0.72 |
| C7 | - | 40.12±1.37 | 53.10±0.64 | 61.15±0.17 |
| C6-1 | - | - | 49.34±0.19 | - |
| C6-2 | - | - | 39.44±1.46 | - |
| C6-3 | - | - | 48.96±0.57 | - |
| C6-4* | - | - | 100.00±1.12 | - |
| C6-5 | - | - | 75.13±0.93 | - |
| C6-6 | - | - | 29.42±0.28 | - |
| C6-4-1 | 57.49±0.77 | 67.23±0.14 | - | - |
| C6-4-2 | 46.93±0.24 | 53.05±0.19 | - | - |
| C6-4-3* | 98.84±0.15 | 100.00±0.10 | - | - |
| C6-4-4 | 70.24±0.18 | 76.56±0.09 | - | - |
| C6-4-5 | 48.26±0.20 | 57.14±0.11 | - | - |
| C5-1 | - | 0 | 0 | 6.58±0.27 |
| C5-2 | - | 4.12±0.25 | 6.24±2.17 | 9.02±1.22 |
| C5-3 | - | 40.42±0.15 | 45.45±0.36 | 50.11±0.45 |
| C5-4 | - | 37.31±1.61 | 41.54±1.03 | 52.03±0.21 |
| C5-5* | - | 65.06±0.72 | 73.14±0.15 | 94.10±1.01 |
| C5-6 | - | 50.03±0.41 | 68.32±0.65 | 79.05±0.29 |
| C5-7 | - | 44.45±0.78 | 51.08±1.21 | 64.12±1.13 |
| C5-8 | - | 29.10±0.91 | 32.11±0.16 | 44.31±0.27 |
| C5-5-1 | 0 | 0 | 0 | - |
| C5-5-2 | 0 | 0 | 12.18±1.73 | - |
| C5-5-3* | 64.34±0.51 | 82.71±0.28 | 97.58±0.39 | - |
| C5-5-4 | 39.08±0.33 | 41.39±1.11 | 49.67±0.20 | - |

The most bioactive subfractions C5 and C6 derived from chloroform-soluble fraction determined for further isolation of bioactive compound.

^a^ positive control

^b^ the BACE- inhibitory activity test not performed at corresponding concentrations.

*represents the bioactive constituents determined for further isolation.

**Table S2.** Summary of antimicrobial inhibitory activity of chloroform- and ethyl acetate-soluble fractions.

| **Materials** | *E. coli* (G-) | *S. aureus* (G+) | *S. enterica* (G-) | *M. luteus* (G+) | *P. multocida* (G-) | *Cytospora sp.* | *P. piricola* | *F. oxysporum f.* sp. *cucumebrium* |
| --- | --- | --- | --- | --- | --- | --- | --- | --- |
| C3-1 | > 15 | > 15 | > 15 | > 15 | > 15 | > 15 | > 15 | > 15 |
| C3-2 | > 15 | > 15 | > 15 | > 15 | > 15 | > 15 | > 15 | > 15 |
| C3-3* | 0.94 | 3.75 | 1.88 | > 15 | 0.46 | 0.46 | > 15 | > 15 |
| C3-4 | > 15 | > 15 | 7.5 | > 15 | > 15 | 15 | > 15 | 7.5 |
| C3-5 | > 15 | > 15 | > 15 | > 15 | > 15 | > 15 | > 15 | > 15 |
| C3-3-1 | > 15 | > 15 | > 15 | > 15 | > 15 | > 15 | > 15 | > 15 |
| C3-3-2* | 0.46 | 3.75 | 0.94 | 15 | 0.46 | 0.46 | 0.94 | 7.5 |
| C3-3-3 | 1.88 | 7.5 | 7.5 | > 15 | 3.75 | > 15 | > 15 | 3.75 |
| C3-3-2-1 | > 3.75 | -^a^ | > 3.75 | - | 3.75 | > 3.75 | > 3.75 | - |
| C3-3-2-2* | 0.46 | - | 0.46 | - | 0.46 | 0.46 | 0.94 | - |
| E8-1 | > 3.75 | - | > 3.75 | - | > 3.75 | - | > 3.75 | - |
| E8-2 | > 3.75 | - | > 3.75 | - | > 3.75 | - | > 3.75 | - |
| E8-3* | 0.94 | - | 0.94 | - | 0.94 | - | 0.94 | - |
| E8-4 | 1.88 | - | > 3.75 | - | > 3.75 | - | 3.75 | - |
| E8-5 | > 3.75 | - | > 3.75 | - | > 3.75 | - | > 3.75 | - |
| E8-6 | > 3.75 | - | > 3.75 | - | > 3.75 | - | > 3.75 | - |
| E8-3-1 | 3.75 | - | > 3.75 | - | 1.88 | - | > 3.75 | - |
| E8-3-2* | 0.94 | - | 0.94 | - | 0.46 | - | 0.94 | - |
| E8-3-3 | 1.88 | - | > 3.75 | - | 3.75 | - | 3.75 | - |

C and E fractions obtained from chloroform- and ethyl acetate-soluble fraction, respectively.

^a^ the antibacterial or antifungal activity tests not performed for corresponding stains.

*represents the bioactive constituents determined for further isolation. The tested concentrations for subfractions ranged from 15.0 to 0.23 and from 3.75 to 0.23 mg/mL based on the amount of isolates.
